# The complete mitochondrial genome of the Chilean endemic frog *Telmatobius chusmisensis* Formas, Cuevas & Nuñez, 2006 (Anura, Telmatobiidae)

**DOI:** 10.1080/23802359.2016.1144097

**Published:** 2016-03-28

**Authors:** Luis Pastenes, Camilo Valdivieso, Talía Del Pozo, Marco A. Méndez

**Affiliations:** a Laboratorio de Genética y Evolución, Departamento de Ciencias Ecológicas, Facultad de Ciencias, Universidad de Chile, Santiago, Chile;; b Laboratorio de Bioinformática y Expresión Génica, INTA, Universidad de Chile, Santiago, Chile;; c Instituto de Ecología y Biodiversidad (IEB), Departamento de Ciencias Ecológicas, Facultad de Ciencias, Universidad de Chile, Santiago, Chile

**Keywords:** Amphibians, high-throughput sequencing, mitogenome, Telmatobius

## Abstract

*Telmatobius chusmisensis* is an endemic frog of northern Chile that is only known at its type locality, Chusmisa. In this study, the complete mitochondrial genome of *Telmatobius chusmisensis* was assembled using high-throughput sequencing data, yielding a circular genome of 19 312 bp with a nucleotide composition of A = 30.8%, C = 24.4%, G = 13.6% and T = 31.2%. Its gene composition and structure were similar to other anuran genomes available: 13 protein-coding genes, two rRNA genes, 22 tRNA genes and the D-loop region. Phylogenetic analysis using Bayesian inference (BI) and maximum likelihood (ML) showed that *Telmatobius chusmisensis*, *T. bolivianus* and *T. vellardi* are a highly supported monophyletic group. This genome information will allow us to gain a better understanding into phylogenetic and phylogeographic relationships in the genus *Telmatobius*.

*Telmatobius chusmisensis* is a frog belonging to the family Telmatobiidae (Frost 2015), endemic of northern Chile, restricted to freshwater streams near the village of Chusmisa, Tarapacá Region (Formas et al. 2006). The classification of its conservation status is Data Deficient by IUCN (Angulo 2008) and Critically Endangered by RCE (Cisternas et al. 2010). Most systematic studies of the genus *Telmatobius* have used mainly morphological characters (e.g. Aguilar & Valencia 2009), whereas studies with molecular markers are scarce (De la Riva et al. 2010; Sáez et al. 2014). To improve our knowledge of this species, we report the complete mitogenome of *T. chusmisensis* (GenBank accession no. KT949346). This information will allow us to gain insight into phylogenetic and phylogeographic relationships that will support further molecular studies to propose conservation measures for this group.

One male adult *T. chusmisensis* was collected from Chusmisa stream (19°41′05.1″S; 69°11′00.6″W) on 10 March 2015 (voucher specimen no. DBGUCH_1503001). Total DNA was extracted from liver tissue using the Dneasy^®^ Tissue & Blood kit (Qiagen, Valencia, CA). The high-throughput sequencing in an Ion Torrent™ PGM^®^ System (Life Technologies, Carlsbad, CA) yielded 1 171 421 reads, which were filtered by quality, trimmed and then mapped to the mitogenome of the closely related species *Telmatobius bolivianus* (GenBank accession no. NC_020002.1) using Geneious^®^ Mapper v8.1.6 (Biomatters Ltd., Auckland, New Zealand).

The complete mitochondrial sequence of *T. chusmisensis* was assembled with 15 023 reads (average coverage 97.7×), revealing a genome of 19 312 bp that shows a non-ambiguous nucleotide composition of A = 30.8%, C = 24.4%, G = 13.6% and T = 31.2%. Gene annotation was performed using Geneious^®^ Annotate and Predict v8.1.6, and confirmed in the DOGMA database (Wyman et al. 2004). Its gene composition and arrangement were similar to the of *T. bolivianus*, consisting of 13 protein-coding genes, two rRNA genes, 22 tRNA genes and the D-loop region. ATG acts mostly as the initial codon in six protein-coding genes, followed by ATC for four protein-coding genes, whereas *nd6* uses ATT and *nd2* and *nd3* use ATA. Eight protein-coding genes use TAA as the stop codon, *cox1* and *nd1* use AGG, and *cox2*, *nd5* and *nd6* use AGA. The start codons of *atp6*, *cox1*, *nd1*, *nd2*, *nd3*, *nd5* and *nd6*, and the stop codons of *cox1*, *cox2*, *cox3*, *nd1*, *nd3* and *nd4l* are different from those of *T. bolivianus*.

Finally, BI and ML trees were constructed using the mitogenomes without the D-loop region of 18 anuran species, including *Pelobates cultripes* and *Pelodytes ibericus* as outgroups. Alignment was performed with MUSCLE v3.5 (Edgar 2004) using default settings. Phylogenetic analyses were performed using resources available in CIPRES Science Gateway v3.3 (Miller et al. 2010). ML tree was inferred using RAxML-HPC v8.2.4 (Stamatakis 2014) with the BlackBox option; BI tree was inferred with MrBayes v3.2.6 (Ronquist & Huelsenbeck 2003) using the XSEDE option. The BI and ML analyses yielded identical topologies (Figure 1). In this phylogeny, *Telmatobius* species formed a monophyletic group, with high support for both ML (bootstrap = 100%) and BI (posterior probabilities = 1.00).

**Figure 1.**
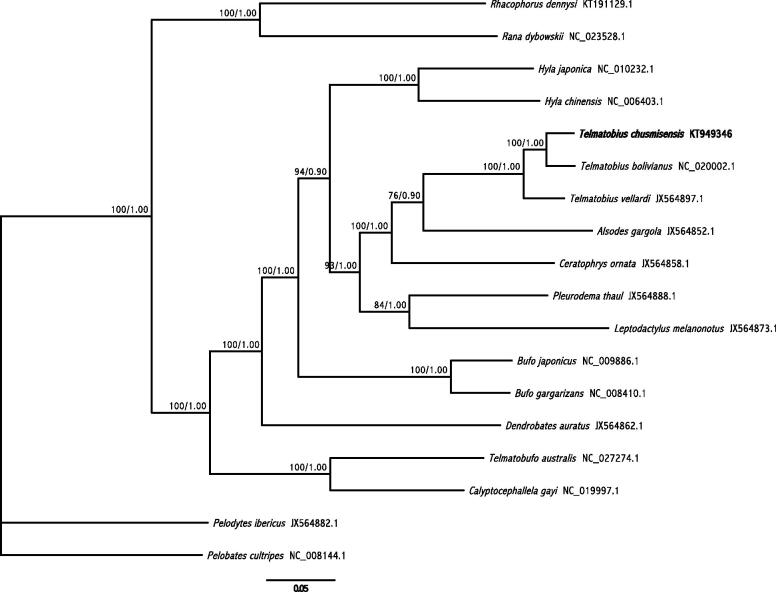
Phylogenetic tree resulting from the ML and Bayesian analyses of 18 mitogenomes without the D-loop region, including two representative Pelabatoidea species that are used as outgroups. GenBank accession numbers follow species names. The numbers along the branches indicate support values (bootstrap/posterior probability value). The position of *Telmatobius chusmisensis* is indicated in bold font.
